# Integrated monitoring: Setting new standards for the next decade of clinical trial practice

**DOI:** 10.4103/2229-3485.76287

**Published:** 2011

**Authors:** Kamala Rai

**Affiliations:** *Head- Clinical Development, International Clinical Research Operations, Novartis Healthcare Pvt. Ltd., Mumbai, Maharashtra, India*

**Keywords:** Conventional monitoring, field monitor, integrated monitoring, trial site

## Abstract

The new age clinical research professional is now geared toward an “integrated monitoring” approach. A number of critical activities at the site level and at the sponsor’s organization need convergence to harness rich dividends in early study start and quick close of the study. The field monitor needs full integration to ensure standard of care, train the site in protocol, select the right site, ensure regulatory support, ensure excellent project management skills, coach, support the logistics team, manage the vendor, ensure good documentation practices, develop patient recruitment and retention, lean the applicable process, as well as ensure effective site management amongst the myriad activities assigned toward developing the drug in the clinic.

## INTRODUCTION

The global clinical research market currently requires contracting with 95,000 clinical trial sites and recruiting approximately 1,200,000 patients/volunteers annually.[1] Owing to the West-to-East drift in clinical trial activity, we witness the emergence of new clinical trial sites and a flurry of activity at clinical research sites, sponsor organizations and regulators at the country level.

The geographical distribution of clinical trial sites and vendors responsible for various services such as IVRS/IWRS, central laboratories for specimen testing and reporting, remote data capture, data management, and other vital services adds to the overall complexity of study management through all phases of the study. Further, the location of the clinical teams and project management teams is also dispersed across the geographies.

As the clinical research market and drug development enters the next decade, increasingly newer markets and opportunities are being explored. Constantly changing regulations across various markets, participation across multinational and multicentric trials, developing newer sites at hitherto unknown clinical trial sites, working on complex protocols with multiple endpoints with complex trial design including the development of Adaptive Trial protocols have increasingly emphasized the role of clinical research professional.

The University of Colorado, Denver, is currently sponsoring a study to evaluate the effectiveness and costs of Remote Monitoring of clinical trials to demonstrate substantial improvements in study monitoring efficiency, effectiveness, and possibly overall costs by Remote Monitoring when compared to the present monitoring plans.[2]

This article will explore the many facets of managing clinical trial protocol at the country operational level to help the new age clinical research professional integrate various aspects of managing the study toward successful closure.

## REDEFINING THE ROLE OF THE FIELD MONITOR – TOWARD “INTEGRATED MONITORING”

Owing to the sudden rise in workload as well as changes in the role of the field monitor, a new breed of monitors termed fully integrated monitors is taking shape. Let us understand the impact of integrated monitoring in shaping the future clinical research practices.

To fully understand the term integrated monitoring, we need to first understand the role of the conventional field monitor.

### Conventional field monitor

The conventional field monitor is hired as a Clinical Research Associate and is trained on the protocol, regulations, as well as therapeutic area by responsible medical advisors. The line manager or the project manager accompanies the field monitor to the trial site and observes the monitoring standards followed. Most often, this training visit also serves to introduce the field monitor to the trial site personnel. Depending on the length of the trial, the field monitor continues to monitor the trial site once in every 4–6 weeks based on the monitoring plan and prepares a report based on the formats described in the sponsor’s standard operating procedure (SOP). The visit report is reviewed by the line manager and open issues are followed until completion. All queries are forwarded by the field monitor to the site for resolution until database lock (DBL) is achieved, and once the DBL is achieved, the clinical trial heads send a congratulatory message.

If the above format of conventional role can lead to possible successful completion of the clinical study, then why is there a need to change the format and style of monitoring?

### Challenges to the conventional model of monitoring

According to industry experts,[3] the conventional field monitor conducts monitoring like a “major surgery” where the field monitor has a detailed checklist of items to evaluate, which could be disruptive and potentially inaccurate since it only reflects a brief snapshot of the overall study.

The role of the field monitor is therefore being redefined across organizations based on improved technology adoption and setting up of lean clinical operation processes. These are meant to reduce stress for the field monitor, decrease costs and increase clinical trial oversight. Some clinical research organizations are adopting a methodology called *Just-in-Time Monitoring* that is dramatically changing the way the field monitors interact with trial sites. The minimally invasive but fully integrated model of monitoring could be far less invasive and disruptive. Incorporation of Remote Monitoring helps in complete data visibility to possibly correct and implement actions, strengthening relationships with trial sites by adopting a more collaborative approach to share feedback more remotely and foster partnership and reduce the cost of conducting trials.

Further need to redefine the role of the field monitor also depends on the increasing development of study protocols based on the Adaptive Trial design.[4]

## COMPONENTS IN ESTABLISHING AN EFFECTIVE CONVERGENT, INTEGRATED MONITORING PLATFORM

Let us re-look some of the components that are absolutely critical while going forward to establish the credentials of a successful clinical research partner and what does this mean for “Integrated Monitoring”.

### Understanding the “burden of disease” and “standard of care”

The field monitor needs to be fully aware of the indication that the protocol aims to address and the overall prevalence and impact for the study management. Knowing this helps the field monitor get insights into understanding the patient definitions described in the study protocol. This is the first step toward developing the fully integrated field monitor. The organization should ensure that the field monitor is tracked for his/her knowledge base on the disease burden as well as the standard of care dispensed internationally and how this varies at the local level. The field monitor should be aware of the prevalent practices for standard of care at the individual site level with reference to standard of care too.

A comprehensive summary needs to be drawn at the country level, especially if there are no national guidelines to manage the indication. The summary of the standards adopted at the individual sites, based on local institutional guidelines, needs to be discussed thoroughly with all field monitors responsible for the site and should be compared to the global standards adopted to manage the indication. Should there be local variations in diagnosing as well as treating the indication, these should be discussed with global teams. A useful tip is to get a global OK on proceeding with the local standard of care despite the variations in comparison to global standard of care.

### Understanding the clinical study protocol as well as all related components impacting study endpoints

For fully integrated monitoring model, the trial sites (atleast few) should have access to the draft protocol and other draft study materials. The field monitor’s ability to negotiate components suggested in the draft protocol with the site personnel ensures a very early buy-in as well as builds excellent rapport and understanding. Further, it prepares the site for the challenges early on and gets a clear understanding of expectations. When the comments suggested by the trial site are rolled up to the final version, the overall ownership of the trial execution at the trial site is realized.

The informed consent document needs to be viewed closely by every stakeholder to ensure that all the principles mandated by regulations are listed in the final document.

All patient materials need to be reviewed and reconciled early on to ensure that the materials are validated, translated locally, and are ready to use prior to study initiation. This can only be made possible at the planning stage through excellent collaboration with the site coordinators, field monitors, project managers, vendors and global teams. The requirements vary institution to institution. A useful tip is to procure the list of clauses in ensuring the development of the patient materials that are mandated as per the local IRB/EC and review the local sponsor SOPs that allow the changes in patient material to be developed and deployed at the trial site.

### Selecting the right site

This is most often based on selection criteria set rigidly and specifically based on the components described in the clinical study protocol.

The challenges in selecting the right site stem solely from the experience and expertise of the field monitor.

The field monitor needs to show vigilance and research acumen in selecting the right site. First, the trial site should also demonstrate willingness, capability in execution, availability of experienced trial personnel, experience in managing the indication and a proven track record in managing the protocol. The experienced trial site needs to confirm the experience of the principal as well as the sub-investigator. A useful tip for the field monitor is to exercise caution in selecting a site that has no prior experience of clinical trial conduct. The organization should ensure that the field monitor assigned to such sites should be well versed in trial execution and conduct as well as experienced, fully qualified and willing to extend his/her expertise to the site.

### Regulatory support and mechanism to ensure safety of trial participants

Most often, the field monitor is confined to the tasks and activities listed across sites assigned to him/her. A useful tip is to share the inputs from regulatory agencies, especially the IRBs/EC, and comments for sites not assigned to them too. Most often, this is noted at an informal setting, but the importance of sharing apprehensions and concerns raised at a IRB/EC at a formal meeting for one site may have a bearing for all sites. The organization should ensure that the there is quick and rapid dissemination of information at the country level across all the field monitors assigned to the sites.

### Excellent project management

The emphasis for excellent project management is absolutely critical for the success of the fully integrated model of monitoring. A successful project manager also ensures that all apprehensions, concerns raised at the global level for timeliness and accuracy of information are allayed, and at the local level, the field monitor and sites are fully aligned to the study protocol and specifications listed.

The project manager demonstrates capability in lining up all the key deliverables, assigns timelines to them based on extensive discussions with teams from clinical groups, operations, vendors, regulatory associates, field monitors and helps in the development and deployment of the monitoring plan.

A useful tip for the field monitor is to develop cordial and professional relationship with the project manager who will serve to co-coach and co-mentor the field monitor assigned to difficult to recruit studies, work with the line manager to develop monitor performance metrics as well as track performance of the sites. The project manager should develop clear differentiation for sites that have recruited and met the targets versus sites that were constantly trained and motivated by the field monitor to meet commitments.

### Regular coaching and mentoring of the field monitors

The clinical research organization should have clear metrics and performance targets in place to provide for incentives and rewards to help establish appropriate coaching and mentoring standards. Most often, the line manager serves as the single point of contact for the field monitor to refer in helping troubleshoot issues at the site, develop and approve travel plan as well as setting up objectives for the field monitor. Most often, the line manager could serve in some organizations to provide project management support too. The organization should therefore ensure that the line manager has sufficient time and expertise to help coach the field monitor in helping raise clinical research standards with new age metrics or reduced source data verification and helping the setting up of trial sites for complex studies. The line manager should be coached by the organization in helping align the overall vision of the organization with those of the territory managers, project managers as well as the field monitors.

### Leveraging key strengths of clinical research professionals

The fully integrated monitor is aware that most clinical research professionals (i.e., investigators, coordinators, and monitors) have varied educational backgrounds and most often rely heavily on guidance from the coordinators they hire and the monitors they encounter.

The organization needs to ensure a mechanism where the training and qualifications of trial personnel are tracked to help standardize research practices at the clinical trial site.

Investigators often take for granted the complexities of drug development, and monitors and coordinators are left without much guidance. The impact of this lack of knowledge and skill on a participating patient’s health and overall care is very real, as is the potential for an adverse impact on the quality of clinical data obtained. It is therefore in the best interests of organization to recruit and hire well-trained researchers, just as it is in the interests of those researchers to achieve as high a degree of training as possible.

### Site performance and track record

The emphasis of establishing and segmenting sites based on several criteria is helping organizations draw up site databases that are ready to use with little lag time in selecting the right site.

The organization should develop clear metrics of high performing sites that are based on actual commitments, time to data entry, time to resolve queries, time to set up the site and overall standards in ensuring patient safety, rights and well-being of trial participants as well as costs per patient per site. The organization should help in disseminating this information to all personnel in the sponsor’s organization responsible for field monitoring. Having all sites performance benchmarked will help the field monitor in successfully deploying the principles of integrated monitoring.

### Availability of trained and experienced field monitors as expert clinical research associates

The fully integrated monitor has all the energies focused external to the clinical research organization, which means that the monitor is aligned completely to the expectations of the site.

Most often, the experienced and expert monitor is rewarded by a line management opportunity that has responsibilities to align the organization internally. While this is crucial in the development of the monitor toward greater responsibilities, the site loses the sponsor’s coach and guide at the site, the trained field monitor. It is time that organizations learn to incentivize the expert monitor at the site and help develop them into successful territory managers and keep them closer to the site.

### Support in logistics and administration

The past few years have witnessed dramatic changes in the role of the field monitor. The field monitor of the 1990s was responsible for setting up and maintaining the sites as well as providing the support in terms of vendor qualification and management, as well as payments, etc. Recently, organizations are investing in setting up new functions within the clinical trial organization. The logistics and administrative function is vital to the overall success of the study. While key tasks have been redistributed from the field monitor’s role to the support role, it is the responsibility of the organization to coach the support staff in helping strengthen overall clinical operations as well as help in vendor qualification and management. In a fully integrated model, role clarity and clear delineation of responsibilities between the in-house support teams and the field monitors will help the sites meet the commitments and raise quality standards.

### Effective vendor management

The drug warehouses, event planners, central laboratories, data management groups, endpoint managers for complex studies, central readers, IVRS/IWRS providers, translators of patient material, and other outsourced entities are all an integral component in trial set-up and maintenance at sites.

The organization should ensure that line managers, project managers, support staff, field monitors are fully aligned and collaborate to the fullest possible extent in working with these groups to understand key deliverables, timelines as well as commitments.

The organization should spend significant time in ensuring that the collaboration amongst the key providers is visible and key deliverables are met.

Continuous coaching and mentoring in conflict resolution should also be an integral part of the plan. One way of ensuring this is to ensure that the victories at the site are often shared with the support staff and vendors, their efforts acknowledged, appreciated and documented.

### Documentation practices at the site

Organizations need to spend time and develop a clear strategy on what is the ideal documentation that could be the basis for regular coaching and mentoring the field monitors and what practices at the site are acceptable and which practices need to have risk mitigation.

The field monitor is confronted with the following challenges:

- Poorly transcribed notes;
- Notes transcribed by a non-medical person (site coordinator who is not trained or qualified);
- Site developed templates based on the visit design of the study that has no formal quality control of listed visit procedures and OK from the sponsor’s team and
- Little clarity on General Physical Examination notes.

A useful tip for the field monitor is review the documentation practice during site selection and help coach and motivate the sites continuously at the study initiation prior to study start to ensure that all source data are captured on the basis of the clarity in agreement with the source data specification sheet.

### Patient recruitment and retention

The field monitor is now confronting acute shortage of experienced trial sites owing to workload as well as competition. Therefore, the sponsor organization must refine performance metrics to include patient recruitment and retention and coach line management as well as project teams to collaborate closely with sites and global teams and develop a set of tools and strategies to help recruit and retain patients.

### Developing new sites

Optimizing territories and developing newer trial sites is again mandatory for every sponsor organization. The organization must invest sufficient time and strategy to help develop new sites by identifying potential sites, helping set up the regulatory framework and SOPs at the site. One useful tip is to incentivize and reward the field monitor for identification of potential site. The field monitor must be given sufficient time and space to help run mock trials and set up mock monitoring visits at the site. Without breaching confidentiality, the field monitor must be encouraged to identify and train potential sites by sharing best and worst practices from other sites.

### Lean processes that ensure rapid initiation and early closure of trial sites

The organization should develop a review mechanism that helps lean all processes with no compromise on the quality and ensuring that all the regulations are adhered to.

### Site management

In the truest sense, the monitors serve as an advocate for the site in the sponsor organization as well as for the sponsor at the site. This is an extremely critical balancing act. For the sponsor, the field monitor selects, sets up, agrees with the site personnel and draws up recruitment targets, develops recruitment and retention strategies, trains coordinators and investigators, reviews and collect research data, serves as patient recruitment consultants, verifies the dispensation of study medications, and reports the ongoing status of research trials.

The monitor serves as a liaison between sites and sponsors and is technically the site manager at individual research centers.

- The focus and emphasis of the monitoring visit and as recommended by the sponsor’s SOP is to review and verify signed, informed consent document in addition to detailed review of all the data collected.
- The data verification process and % of Source Data Verification (SDV) varies across the sites based on the experience, expertise and overall monitoring plan developed for the study.
- The monitor must be coached to freely exercise the option of scheduling a remote monitoring visit or a physical site visit.
- Useful tip for the monitor is to adopt the “continuous data flow” strategy where the data are continuously reviewed during the entire duration of the trial and the data are entered in a timely and consistent manner as mandated by the sponsor SOPs.
- The other aspects to be noted are the unblinding procedures and understanding clearly the role of the unblinded monitor and the pharmacist. The sponsor organization should ensure sufficient training and role clarity in ensuring the effectiveness of the unblinded monitor/pharmacist should the study mandate such monitoring.
- The monitoring standards should help define clear guidelines for physically counting and comparing all the remaining study drugs with the site’s dispensation logs.
- Further, the sponsor’s organization should establish standard in training the monitor to match study participants’ medical histories with the inclusion and exclusion criteria relevant for a specified trial in ensuring patient eligibility.
- The sponsor should also document the training of the field monitor in “Risk Management Plan” in ensuring effective dissemination of safety data and reporting of adverse events in a timely manner.
- The fully integrated monitor while working closely with the in-house teams of line managers, support teams, global teams and site personnel should learn to take a quick global “snapshot” of how each patient is progressing during the trial. The monitor should be trained and developed to ensure that patients in the trial are receiving adequate patient care. This can be established by closely networking with medical monitors/advisors assigned to the study. Further, they should be experienced to comment if the collected data are coherent.
- The monitor should summarize effectively and conclusively if the trial is progressing satisfactorily.
- The monitor should have developed an escalation plan to discuss the concerns with investigators, in-house teams as well as coordinators.
- The monitor has a critical role to play in ensuring that the CRF data are entered according to the rigid requirements of a clinical database. Monitors must be sufficiently familiar with both the clinical database and the CRF to help sites capture patient data in a way that is accurate but also meets the electronic specifications. The monitor must learn to adopt technology more effectively.

Monitors must ensure that the documentation of issues should include only facts and no guesses, no thoughts and no theories. This is extremely important as these reports become a part of a sponsor’s official study file, and as a result, monitors must word these reports carefully and seriously.

### Soft skills

- A fully integrated monitor helps develop strong communication links at the research site. Further, the monitor is trained by the sponsor to observe everything that occurs at the site. This could include the number of patients in the waiting room, site personnel attrition, stressed staff at the site, etc.
- The monitor must be trained to bring in site personnel issues quickly to the investigator. Any issue involving investigator negligence and fraud must be brought to the attention of the sponsor. The practice to be followed is to observe keenly and listen intently to all that is happening at the trial site and to document all that is observed. Further, these issues could be discussed with the trial personnel and the investigator. The monitor must also be trained to escalate legitimate issues to in-house teams responsible for the study.
- The sponsor organization needs to orient the monitor to help juggle quite a number of tasks related to regulatory and protocol requirements, study conduct issues, supervisory concerns, and data management matters in addition to coordinating meetings, being aware of the time zone difference.
- The monitor must be trained to attend teleconferences, schedule meetings, document minutes of such meetings and participate proactively.

## CONCLUSIONS

The past few years have witnessed an increasing inflow of clinical trial workload in almost all therapeutic areas, thus seeking definite intervention in setting up trial sites for complex, difficult to recruit studies as well. This has posed phenomenal burden on the work-life of the field monitor. Toward this, organizations have redefined job roles and delineated responsibilities to help support the sites as well as the field monitor. Organizations need to understand the responsibility of the field monitor in not just monitoring trial patient data at the site, but to grant sufficient authority to the field monitor to work with various groups in-house to help deliver the best clinical trial package to the site and also be fully equipped to keep in-house teams informed and kept abreast of all activities that are impacting trial performance at the site.

The new age field monitor understands the power of “integration” and helps the organization to “converge at the site” and engages continuously with all clinical research professionals responsible for trial execution and conduct.
